# A treatment approach for couples with disrupted sperm DNA integrity and recurrent ART failure

**DOI:** 10.1007/s10815-019-01543-5

**Published:** 2019-08-16

**Authors:** Alessandra Parrella, Derek Keating, Stephanie Cheung, Philip Xie, Joshua D. Stewart, Zev Rosenwaks, Gianpiero D. Palermo

**Affiliations:** The Ronald O. Perelman and Claudia Cohen Center for Reproductive Medicine, Weill Cornell Medicine, New York, NY 10021 USA

**Keywords:** Assisted reproductive technologies, Sperm chromatin fragmentation, Density gradient selection, Microfluidic chamber, ICSI

## Abstract

**Objective:**

To test a novel method to select spermatozoa with high chromatin integrity.

**Design:**

Specimens with high sperm chromatin fragmentation (SCF) were selected by density gradient selection (DGS) and microfluidic sperm sorting (MSS).

**Setting:**

Academic medical center.

**Patient(s):**

Ejaculates from consenting men were processed by DGS/MSS. Couples underwent ICSI cycles with spermatozoa processed by DGS/MSS. Clinical outcomes were evaluated after embryo transfer.

**Intervention(s):**

SCF was measured by TUNEL. ICSI with spermatozoa selected by DGS and MSS was performed.

**Main outcome measure(s):**

Fertilization, embryo implantation, and pregnancy outcomes were compared between DGS and MSS.

**Result(s):**

A total of 23 men had an average SCF of 20.7 ± 10%. After DGS and MSS, the SCF was 12.5 ± 5% and 1.8 ± 1%, respectively. In couples who underwent ICSI, the average SCF was 28.8 ± 9%, which fell to 21.0 ± 9% after DGS and 1.3 ± 0.7% after MSS. Four couples underwent 11 ICSI cycles with DGS and achieved one (25%) pregnancy that resulted in pregnancy loss. In four subsequent ICSI cycles with MSS, an ongoing clinical pregnancy rate of 50% was achieved. Five additional couples underwent 12 cycles of ICSI with DGS. After preimplantation genetic testing for aneuploidy, 30.3% of the embryos were euploid. One pregnancy was achieved, resulting in pregnancy loss. With MSS, 31.5% of the embryos were euploid and 4 couples obtained a pregnancy. Finally, sixteen couples underwent 20 ICSI cycles solely with MSS at our center. Of these couples, 8 had failed 13 ICSI cycles with DGS elsewhere. These couples achieved an overall implantation of 34.5% (10/29) and a pregnancy rate of 58.8% (10/17).

**Conclusion(s):**

Microfluidic selection yielded spermatozoa with optimal genomic integrity and improved chances of obtaining a euploid conceptus.

## Introduction

Infertility affects 12–18% of couples in the USA of reproductive age [1]. Up to 15% of all infertility cases may remain unexplained, despite involving couples in which no obvious factor can be traced to either partner [2]. The reproductive treatment approach for these couples is somewhat incremental, beginning with ovulation monitoring in conjunction with timed intercourse or intrauterine insemination. If these initial treatments fail, a more invasive technique is used such as in vitro fertilization (IVF) or intracytoplasmic sperm injection (ICSI) [3]. When a specific cause for the infertility cannot be identified, a subtle male factor may be presumed. Indeed, up to 11% of men with a normal semen analysis have a noticeable sperm chromatin fragmentation (SCF), which may affect up to 5% of men with semen parameters above the 50th percentile [4, 5]. In addition, an inverse correlation between SCF and sperm motility has been shown [6]; therefore, these men are better suited for ICSI insemination in which sufficiently motile sperm cells are identified under direct visualization [7, 8]. It should be noted that a mild degree of sperm DNA fragmentation can be prevented by ooplasmic repair mechanisms [9–11], which have been confirmed by studies assessing the ability of donor eggs to repair spermatozoa with compromised chromatin [12]. Nevertheless, this ooplasmic intervention can be defective in certain couples, particularly in cases where the woman is of advanced reproductive age.

After observing couples with unexplained infertility after a failed IUI, we designed a reproductive treatment algorithm based on a sperm DNA fragmentation assay [13]. According to this algorithm, if the male gamete chromatin integrity is confirmed, the couple can proceed to standard in vitro insemination. Conversely, if an abnormal SCF is reported, the couple is offered ICSI at the outset, utilizing ejaculated spermatozoa. ICSI would also be offered to men who, in spite of having spermatozoa with healthy chromatin, failed to achieve a pregnancy with standard in vitro insemination. Finally, for cases in which a pregnancy has not been achieved even with ICSI, men are counseled by a reproductive urologist and offered a cycle of ICSI using spermatozoa retrieved directly from the testicle [13, 14], where SCF appears to be consistently lower [15, 16].

Testicular biopsy, however, is a radical and invasive procedure that may result in scarring of the seminiferous tubules with consequent overall impairment of spermatogenesis. Moreover, the correlation between motility, particularly the progressive type, and sperm chromatin integrity prompted us to investigate alternative and more conservative procedures. Among various options, microfluidic devices have been proposed to select more suitable spermatozoa [15, 17–20] with higher chromatin integrity and presumably greater fertilizing potential and an improved ability to support embryonic development. Since these novel devices can be sophisticated, expensive, and at times cumbersome, simpler methods have been tested. A recent report has shown the ability, and presumed clinical safety, of a chip sperm sorting device to select directly from the raw semen highly motile spermatozoa characterized by superior chromatin integrity [21].

In this investigation, SCF was assessed on aliquots of sperm specimens selected by a commercial microfluidic sperm sorting device (ZyMōt™ Multi device; DxNow, Gaithersburg, MD) and standard density gradient in comparison with raw semen. In addition, in couples with recurrent assisted reproductive technology (ART) failure and male partners with high sperm DNA fragmentation, we assessed the fertilization and implantation potential, as well as the ability to support embryonic development, of spermatozoa processed by microfluidic sperm sorting as compared with conventional density gradient centrifugation.

## Materials and methods

### Patients

This study was approved by the Institutional Review Board at Weill Cornell Medicine (IRB No. 1705018205, IRB No. 1210013187, and IRB No. 0712009553), and all couples were counseled and signed informed consent forms in order to participate. From January 2017, a total of 48 patients enrolled in this study. Twenty-three men underwent an initial semen analysis and an additional 25 couples underwent ART cycles.

### Semen specimen collection, classification, and selection

Fresh semen specimens with an average abstinence of 2–5 days were processed for standard semen analysis. Specimens were allowed to liquefy for at least 15 min at 37 °C prior to analysis. A small quantity (5 μL) of raw sample was loaded onto a Makler chamber. Additional 5-μL aliquots of these raw samples were smeared on a morphology slide and evaluated. Volume, concentration, motility, and morphology parameters were appraised according to WHO criteria [2]. Raw specimens were allocated in 2 aliquots for processing by density gradient centrifugation and microfluidic sperm sorting.

#### Density gradient selection (DGS)

After the addition of HEPES-buffered human tubal fluid medium (H-HTF; Irvine Scientific, CA, USA) supplemented with human serum albumin (HSA solution G Series culture media; Vitrolife, Goteborg, Sweden), samples were centrifuged at 600*g* for 10 min. The pellet was loaded onto 1 mL of 90% density gradient (Enhance-S Plus Cell Isolation Media, 90%; Vitrolife), followed by centrifugation at 300*g* for 10 min. Using a glass pipette, the selected sperm pellet was resuspended in medium and centrifuged at 600*g* for 10 min to remove silica gel particles. The supernatant was discarded, and 5 μL of the final sample was reassessed in the Makler chamber for final semen parameters. After preparation, 5 μL of the final sample was reassessed in the Makler chamber for the detection of concentration, motility, and morphology.

#### Microfluidic sperm sorting (MSS)

MSS was performed using a commercial single-use device (ZyMōt™ Multi (850 μL) device; DxNow, Gaithersburg, MD). The chip is made of poly(methyl methacrylate) (PMMA) with a single inlet channel that leads to a collection chamber separated by an 8-μm porous microfilter that allows spermatozoa with the highest progressive motility to reach the upper outlet chamber for specimen extraction. For ICSI preparation, 850 μL of sample was loaded into the inlet of the device. An equivalent amount of HTF medium was loaded on top of the membrane. The loaded device was placed in a humidified incubator at 37 °C for 30 min. Following incubation, spermatozoa were retrieved using the outlet port with a syringe (Fig. 1). The sorted suspension was reassessed for concentration, motility, morphology, and DNA fragmentation.

### Sperm DNA fragmentation assay

To assess sperm chromatin integrity, 20 μL of sample before and after each selection method was smeared on a slide. Using the samples before and after each selection method, terminal deoxynucleotidyl transferase–mediated deoxyuridine triphosphate-fluorescein nick end labeling (TUNEL) assay protocol was used as previously described [6]. In brief, a commercially available kit was used to perform the assay (in situ cell death detection kit; Roche Diagnostics, Rotkreuz, Switzerland). After production, raw semen samples were allowed to liquefy in an oven at 37 °C for 15 min. Slides were smeared with 5 μL of the semen sample and left to dry overnight. The slides were placed in 4% paraformaldehyde for 1 h to allow for fixation of the samples. Slides were then washed in PBS and left to dry overnight once more. Slides were immersed for 2 min at 4 °C in a permeabilization solution with 0.1% Triton X-100 and 0.1% sodium citrate in PBS. The enzyme and label solutions were applied to the slides according to the dilutions specified in the kit protocol and left to incubate under coverslips in a humidified chamber at 37 °C for 1 h. Slides were subsequently washed thrice in PBS, and DAPI/Antifade solution was added in order to visualize sperm nuclei, which were observed under a fluorescent microscope for signals indicating DNA breakage. A minimum of 500 spermatozoa was assessed per patient; a sperm DNA fragmentation (SDF) of ≤ 15% was considered normal.

### Ovarian superovulation and oocyte collection

Only couples with normal BMI and no history of smoking, excess drinking, or use of recreational drugs were included. All patients included were Caucasian and had comparable durations and indications of infertility. The average AMH of the female partner was 3.2 ± 3 ng/mL, the average FSH was 5.3 ± 4 IU/mL, and the average peak estradiol for ICSI stimulation was 3066.3 ± 1731 pg/mL.

A similar superovulation protocol was used for all couples. Patients were treated with daily gonadotropins (Follistim; Merck, Kenilworth, NJ, USA; Gonal-F; EMD-Serono, Geneva, Switzerland; and/or Menopur; Ferring Pharmaceuticals Inc, Parsippany, NJ, USA). Precocious ovulation was prevented by GnRH antagonist administration (Ganirelix acetate; Merck, Kenilworth, NJ, USA; or Cetrotide; EMD-Serono Inc., Rockland, MA, USA). The trigger for final oocyte maturation with human chorionic gonadotropin (Pregnyl, Merck) was administered when the two leading follicles reached a diameter of ≥ 17 mm [22, 23]. Transvaginal oocyte retrieval was performed under conscious sedation 35–37 h after hCG administration.

Cumulus-corona cells were removed by exposure to medium containing 40 IU/mL hyaluronidase (Cumulase; Halozyme Therapeutics, Inc., San Diego, CA) [24] and incubated 1–2 h prior to ICSI.

### Embryo culture and morphologic and cytogenetic evaluation

Embryo evaluation, criteria, and the biopsy procedure have been described previously [25]. Day-3 embryos considered good quality had morphologic grades of 1–2 (≥ 8 cells with even or slightly uneven blastomere expansion and ≤ 20% fragmentation). Day-5 good-quality embryos had the following morphologic grades: blastocele, 1–3 (degree of expansion ≥ 50% the volume of the embryo); inner cell mass, A–B (clear inner cell mass with healthy cells); and trophectoderm, A–B (healthy cells).

The biopsy procedure was performed on day 5 as follows: The embryo was positioned by a holding a pipette while laser pulses (ZI-LOS-tk Laser) were used to create an opening in the zona pellucida to allow for aspiration of three to seven trophoblastic cells with a 20-μm-diameter biopsy pipette. Cells were transferred in 200 μL of polymerase chain reaction tubes with 2 μL of lysis buffer and analyzed by array comparative genome hybridization (Bluegnome 24SureV3 chip; Illumina, San Diego, CA). Biopsied embryos were rinsed in a culture buffer and then vitrified [26] until the cytogenetic results were available and patient synchronization was achieved for embryo transfer.

### Embryo transfer and pregnancy assessment

Patients received intramuscular progesterone supplementation (50 mg daily) starting 1 day after retrieval. Couples underwent a fresh embryo transfer on day 3 or 5 according to the developmental characteristics of the embryo. Couples that underwent preimplantation genetic testing for aneuploidy (PGT-A) had euploid embryo transfers in natural or programmed FET cycles [27]. Endometrial lining was at least 7 mm in order for a patient to be considered for an embryo transfer. Serum βhCG levels were measured 14 days after retrieval. Clinical pregnancy was defined as the detection of an intrauterine implantation sac with fetal heart activity on ultrasound.

### Statistical analysis

Continuous variables were described as a mean ± SD (standard deviation). The Friedman test was used when the data was paired and had 3 or more treatment groups. The Kruskal-Wallis test was used when there were 3 or more treatment groups, but the data was unpaired. The Mann-Whitney test was used when data was unpaired and two treatments were assessed at a time. Variables in this category include volume, concentration, motility, normal forms, and DNA fragmentation. For categorical variables, represented as a percentage, the chi-square test was performed to test for relationships between the two groups. Variables in this category are fertilization, clinical pregnancy, and pregnancy loss. A *P* value was reported when the differences between the groups were statistically significant (at a threshold of 0.05). R software (R Foundation for Statistical Computing; Vienna, Austria) was used to perform all statistical analyses.

## Results

**Fig. 2:**
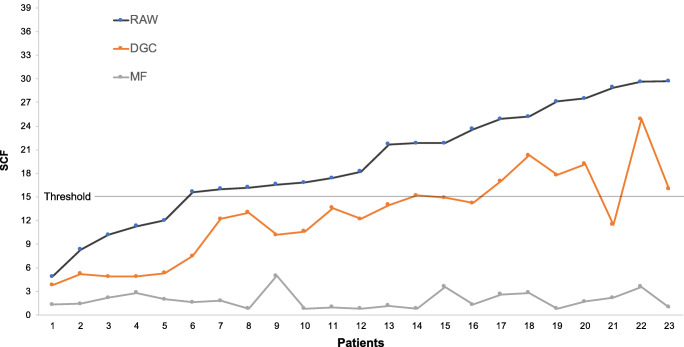
Ranked SCF assessment carried out in 23 men and compared to the level of chromatin integrity achieved after DGS and MSS

To test the efficacy of this novel selection method, particularly its ability to identify the highest progressively motile spermatozoa characterized by optimal chromatin integrity, we screened 48 men for SCF levels using raw semen, and after processing by DGS and MSS. Semen specimens were obtained from 23 men and SCF was assessed following 3 aliquots from each specimen: raw, density gradient, and microfluidic processing. Although the sperm concentration following MSS decreased (*P* < 0.0001), motility and normal morphology significantly improved (*P* < 0.0001). Furthermore, sperm DNA fragmentation decreased from 20.7% in the raw specimen to 1.8% after MSS (*P* < 0.0001; Table 1 and Fig. 2).

**Table 1:** Parameters and SCF values in aliquots of specimen processed by density gradient and microfluidics in comparison to raw semen of 23 men

Parameters (mean ± SD)	Selection			
	Raw	Density gradient	Microfluidics	*P* value
Volume (mL)	2.9 ± 1.7*^†^	0.5 ± 0.02*	0.4 ± 0.08^†^	< 0.0001
Concentration (× 10^6^/mL)	61.8 ± 35*^†^	40.5 ± 25*^‡^	15.3 ± 12^†‡^	< 0.0001
Motility (%)	34.1 ± 14*^†^	65.4 ± 31.4*^‡^	96 ± 11^†‡^	< 0.0001
Progressive (%)	30.1 ± 14*^†^	67.2 ± 29.7*^‡^	97.6 ± 2^†‡^	< 0.0001
Non-progressive (%)	4.3 ± 2*^†^	2.1 ± 1.5*^‡^	0.08 ± 0.2^†‡^	< 0.0001
Morphology (%)	2.5 ± 0.8^†^	2.6 ± 1^‡^	4.0 ± 0.7^†‡^	< 0.0001
DNA fragmentation (%)	20.7 ± 10*^†^	12.5 ± 5*^‡^	1.8 ± 1^†‡^	< 0.0001

*^‡^*P* < 0.001

^†^*P* < 0.0002

This table describes the semen characteristics (including the DNA fragmentation) of the raw specimen and compared them with those obtained after density gradient and microfluidic selection. Statistical evaluation is depicted comparing the values between raw specimen and each selection method

Encouraged by these findings, we then applied the same method to couples who underwent ICSI. This cohort included 25 couples (maternal age, 37.3 ± 6 years; paternal age, 44.4 ± 10 years). The superovulation protocol of the female partners and the couples’ age, BMI, history of infertility, ethnicity, indication to infertility, smoking and drinking habits, and the use of recreational drugs were comparable or controlled for among these 25 patients.

Semen characteristics and SCF were evaluated before and after DGS and MSS processing for each patient, who therefore served as their own control (Table 2). While there was a decrease in concentration (*P* < 0.0001), a concurrent improvement in total motility, progressive motility, and morphology of the microfluidic sorted aliquots was seen (*P* < 0.0001). SCF dropped below threshold after density gradient selection (*P* < 0.0001) and fell dramatically after microfluidic sorting (*P* < 0.0001; Table 2).

**Table 2:** Parameters and SCF values in aliquots of specimen processed by density gradient and microfluidics in comparison to raw semen of the male partner of 25 couples undergoing ICSI

Parameters (mean ± SD)	Selection			*P* value
	Raw	Density gradient	Microfluidics	
Volume (mL)	1.9 ± 1.0*^†^	0.5 ± 0.1*^‡^	0.4 ± 0.05^†‡^	< 0.0001
Concentration (× 10^6^/mL)	32.7 ± 34*^†^	25.8 ± 30*^‡^	7.5 ± 11^†‡^	< 0.0001
Motility (%)	30.2 ± 13*^†^	53.1 ± 30*^‡^	98 ± 3^†‡^	< 0.0001
Progressive (%)	26.0 ± 13*^†^	51.2 ± 31*^‡^	97.7 ± 3^†‡^	< 0.0001
Non-progressive (%)	4.1 ± 3*^†^	1.8 ± 2.2*^‡^	0.2 ± 0.8^†‡^	< 0.0001
Morphology (%)	2.1 ± 0.7^†^	2.3 ± 1^‡^	3 ± 1^†‡^	< 0.0001
DNA fragmentation (%)	28.8 ± 9*^†^	21 ± 9*^‡^	1.3 ± 0.7^†‡^	< 0.0001

*^‡^*P* < 0.05

^†^*P* < 0.001

This table describes the semen characteristics (including the DNA fragmentation) of the raw specimen and compared them with those obtained after density gradient and microfluidic selection. Statistical evaluation is depicted comparing the values between raw specimen and each selection method

**Fig. 1:**
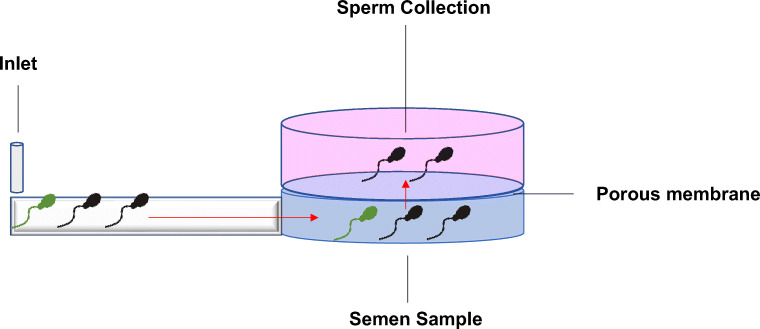
Raw semen samples are loaded into the bottom chamber of the microfluidic device through an inlet and incubated. Spermatozoa with the highest progressive motility and superior chromatin integrity are able to pass through the porous membrane into the upper chamber, where they are collected after incubation

Next, to compare the impact of selected spermatozoa on embryo developmental competence, we treated four couples with male partners who had a remarkably elevated SCF in their ejaculate (34.1%). The semen parameters for microfluidics as well as the SCF are depicted in Table 3. In this group of men, chromatin integrity improved dramatically (1.6%) when spermatozoa went through the microfluidic chamber (*P* < 0.02) (Fig. 1). There were also significant improvements in motility (*P* < 0.02), with a minor improvement in spermatozoa morphology. These couples (maternal age, 35.9 ± 4 years; paternal age, 40.0 ± 6 years) underwent 11 ICSI cycles with DGS and achieved 26.3% good-quality embryos with a fertilization rate of 59% that yielded a biochemical pregnancy and a pregnancy loss at 7 weeks. Once MSS spermatozoa were used in four cycles, a comparable fertilization rate (61.2%) was achieved with a trend toward a larger proportion of good-quality embryos (57.1%) that resulted in an implantation rate of 25% and a clinical pregnancy rate of 50% (Table 4).

**Table 3:** Semen parameters and SCF of couples that underwent ICSI by DGS processing and subsequently by MSS

	Selection			*P* value
	Raw	Density gradient*	Microfluidics	
Volume (mL)	1.4 ± 2	0.2 ± 0.1	0.4 ± 0.1	< 0.05
Concentration (× 10^6^/mL)	2.0 ± 5	3.1 ± 5	0.12 ± 0.1	< 0.05
Motility (%)	3.6 ± 12	10 ± 13	96.5 ± 5	< 0.02
Morphology (%)	1.2 ± 0.5	1.4 ± 0.5	2.0 ± 0	NS
DNA fragmentation (%)	34.1 ± 9	26 ± 4	1.6 ± 0.7	< 0.02

*3 samples were processed with centrifugation method due to low concentration

This table describes the semen characteristics (including the DNA fragmentation) of the raw specimen and compared them with those obtained after density gradient and microfluidic selection. Statistical evaluation is depicted comparing the values between raw specimen and each selection method

**Table 4:** Clinical outcome of couples that underwent ICSI by DGS processing and subsequently by MSS

Number of (%)	Selection		*P* value
	Density gradient	Microfluidics	
Patients	4		
Cycles	11	4	
Injected oocytes (*M* ± SD)	7.5 ± 5	12.2 ± 6	NS
Fertilization rate (2PN)	49/83 (59.0)	30/49 (61.2)	NS
Embryo transfers	4	4	
Embryos transferred	19	8	
Good quality	5/19 (26.3)	4/7 (57.1)	NS
Pregnancy with			
+ βhcg	2/4 (50.0)	2/4 (50.0)	NS
+ FHB	1/4 (25.0)	2/4 (50.0)	NS
Implantation	1/19 (5.2)	2/8 (25.0)	NS
Clinical pregnancy rate	1/4 (25.0)	2/4 (50.0)	NS
Pregnancy loss	1/1 (100.0)	0/4 (0.0)	NS
Ongoing/delivered	0/4 (0.0)	2/4 (50.0)	NS

This table describes the clinical outcome of 4 couples that underwent ICSI with spermatozoa processed by density gradient and later by microfluidics. Statistical analysis is presented

We then included 5 couples (maternal age, 36.4 ± 5 years; paternal age, 36.7 ± 7 years) who planned to have their embryos screened by PGT-A, following ICSI. When sperm samples from the male partners in this group were processed by DGC, the motility significantly improved (28% raw vs 51% DGS; *P* < 0.05), while sperm morphology and SCF remained comparable. After using MSS, there was a clear and net improvement in motility (28% raw vs 99% MSS; *P* < 0.0001) and SCF (19.2% raw vs 1.5% MSS; *P* < 0.01) (Table 5). There was also a reduction in concentration, but this was not detrimental to ICSI insemination. These couples underwent 12 ICSI cycles where semen specimens were processed by DGS, resulting in a satisfactory fertilization rate (78%) and an adequate number (23/33, 69.7%) of morphologically good-quality embryos. PGT-A identified 10 euploid embryos that generated a pregnancy, which did not go to term. In the 9 cycles in which MSS was used, there was a comparable fertilization rate (72.8%) as well as number (27/38, 71%) of good-quality embryos of which half were confirmed euploid. Thus far, 4 embryos were transferred in four couples, all of which implanted and resulted in ongoing clinical pregnancies (*P* < 0.01; Table 6).

**Table 5:** Parameters of SCF of sperm specimen processed by density gradient or microfluidics of couples undergoing ICSI

Parameters (mean ± SD)	Selection			*P* value
	Raw	Density gradient	Microfluidics	
Volume (mL)	2.2 ± 1.7*^†^	0.5 ± 0.4*	0.5 ± 0.2^†^	< 0.001
Concentration (× 10^6^/mL)	38 ± 42	2.6 ± 2	2.8 ± 4.4	NS
Motility (%)	28 ± 22*^†^	51 ± 30*^‡^	99 ± 1^†‡^	< 0.0001
Morphology (%)	2.6 ± 0.9	3.0 ± 1	3.8 ± 0.8	NS
DNA fragmentation (%)	19.2 ± 5^†^	14.2 ± 4^‡^	1.5 ± 1.4^†‡^	< 0.01

*P < 0.05

^†‡^*P* < 0.02

This table describes the semen characteristics (including the DNA fragmentation) of the raw specimen and compared them with those obtained after density gradient and microfluidic selection. Statistical evaluation is depicted comparing the values between raw specimen and each selection method

**Table 6:** Clinical outcome of couples who underwent ICSI with DGS and subsequently MSS and received a thawed PGT-A screen embryo transfer

Number of (%)	Selection		*P* value
	Density gradient	Microfluidics	
Patient	5		
Cycles	12	9	
Injected oocytes (*M* ± SD)	8.1 ± 4	9 ± 4	NS
Fertilization rate (2PN) (%)	76/97 (78%)	59/81 (72.8%)	NS
Embryos screened	33	38	
Good quality	23/33 (69.7%)	27/38 (71.0%)	NS
Euploid	10/33 (30.3%)	12/38 (31.5%)	NS
Embryos transferred after PGT-A	10	4	NS
Pregnancy with			
+ βhcg (%)	1/5 (20%)	4/4 (100%)	< 0.05
+ FhB	1/5 (20%)	4/4 (100%)	< 0.05
Implantation	1/10 (10%)	4/4 (100%)	< 0.01
Clinical pregnancy rate	1/5 (20%)	4/4 (100%)	< 0.05
Pregnancy loss	1/1 (100%)	0/4 (0%)	NS
Ongoing/delivered (%)	0/5 (0%)	4/4 (100%)	< 0.01

This table describes the clinical outcome after PGT-A of 5 couples that underwent ICSI with spermatozoa processed by density gradient and later by microfluidics. Statistical analysis is presented

Finally, 16 couples that had been treated at other clinics and failed to achieve a pregnancy underwent ICSI solely with MSS at our center. In a preliminary comparison of semen parameters following DGS and MSS, motility (59.3% DGS vs 97.6% MSS; *P* < 0.02) and morphology (2.1% DGS vs 3.3% MSS; *P* < 0.05) increased dramatically while SCF (18.5% DGS vs 1.2% MSS; *P* < 0.02) decreased (Table 7). Of these couples, 8 underwent 13 ICSI cycles elsewhere, yielding a fertilization rate of 74.1%, but no pregnancies were reported. A total of 9 couples underwent 12 ICSI cycles with MSS and fresh embryo transfer, with a 74% fertilization rate and 83.3% (20/24) good-quality embryos. These cycles resulted in 25% (6/24) implantation and 50% (6/12; *P* < 0.05) clinical pregnancy rate (Table 8). The remaining 7 couples who underwent 8 ICSI cycles with MSS opted for PGT-A due to their history of having all chromosomally abnormal embryos. In these couples, a fertilization rate of 78.4% (62/79) progressed to 62.1% (23/79) good-quality embryos of which 51% (19/37; *P* < 0.001) were euploid. Five embryos were transferred in four patients who achieved an 80% (4/5; *P* < 0.01) implantation and clinical pregnancy rate (*P* = 0.01) (Table 8).

**Table 7:** Parameters of SCF of sperm specimen of 16 couples treated solely by microfluidic sperm selection at our center

	Selection			*P* value
	Raw	Density gradient	Microfluidics	
Volume (mL)	1.8 ± 1*^†^	0.5 ± 0.02*	0.5 ± 0.05^†^	< 0.05
Concentration (× 10^6^)	40 ± 39^†^	32.3 ± 36^‡^	9.1 ± 13^†‡^	< 0.05
Motility (%)	32 ± 12*^†^	59.3 ± 29*^‡^	97.6 ± 2^†‡^	< 0.02
Morphology (%)	2.1 ± 1^†^	2.1 ± 1^‡^	3.3 ± 1.1^†‡^	< 0.05
DNA fragmentation %)	29 ± 9*^†^	18.5 ± 11*^‡^	1.2 ± 0.4^†‡^	< 0.02

*^‡^*P* < 0.05

^†^*P* < 0.005

This table describes the semen characteristics (including the DNA fragmentation) of the raw specimen and compared them with those obtained after density gradient and microfluidic selection. Statistical evaluation is depicted comparing the values between raw specimen and each selection method

**Table 8:** Reproductive history of 16 couples treated solely by microfluidic sperm selection at our center

	Microfluidics				
	DGS elsewhere^a^	Fresh embryo transfer^b^	PGT-A embryo transfer^c^	*P* value^a,b^	*P* value^a,c^
Patients	8	9	7		
Cycles	13	12	8		
Injected oocytes (*M* ± SD)	8.3 ± 9	10 ± 8	8.7 ± 5	NS	NS
Injected oocytes	116	123	79		
Fertilization rate (2PN)	86/116 (74.1%)	91/123 (74%)	62/79 (78.4%)	NS	NS
Embryos screened	12	–	37		
Good quality	N/A	20/24 (83.3%)	23/37 (62.1%)		
Euploid	0/12 (0%)	–	19/37 (51%)	–	< 0.001
Cycle w/ transfer	7	12	5		
Embryos transferred	8	24	5		
Pregnancy with					
+ βhcg	0/7 (0%)	7/12 (58.3%)	4/5 (80%)	< 0.05	0.01
+ FhB	0/7 (0%)	6/12 (50%)	4/5 (80%)	< 0.05	0.01
Implantation	0/8 (0%)	6/24 (25%)	4/5 (80%)	NS	< 0.01
Clinical pregnancy rate	0/7 (0%)	6/12 (50%)	4/5 (80%)	< 0.05	0.01
Pregnancy loss	–	2/6 (33%)	0/4 (0%)	–	–
Ongoing/delivered	0/6 (0%)	4/12 (33%)	4/5 (80%)	NS	0.01

This table describes the clinical outcome of couples that underwent ICSI at our center with spermatozoa processed by microfluidics. Results are distinguished between couples that underwent a fresh embryo transfer or with PGT-A selection. Historical cycles performed elsewhere with density gradient selection are also reported for comparison. Statistical analysis is presented

## Discussion

Unexplained infertility may be attributed to a subtle male factor linked to compromised sperm DNA integrity. During ICSI, it is possible to select the most progressively motile spermatozoa that therefore have the highest chromatin integrity [6]. Moreover, it has been known for some time that once the male genome penetrates the oocyte, ooplasmic mechanisms decondense sperm chromatin and reconstitute nucleosomes by replacing protamine with histones while processing and repairing DNA strands [9, 10, 28, 29]. Therefore, a young oocyte along with the selection of the most motile spermatozoa for ICSI insemination may prevent these male genomic defects [10, 11, 29].

In the most persistent cases of male factor infertility, utilizing spermatozoa retrieved directly from the testis, presumed to retain superior chromatin integrity, has been proposed [13, 14, 30]. However, this technique is not without drawbacks, as gametes retrieved directly from the seminiferous tubules can have lower fertilization capacity, which is typical of testicular spermatozoa. Moreover, microsurgical testicular biopsy, although focused on individual tubules, is an invasive and costly procedure that may be associated with damage of the surrounding tissue with consequent scarring, requiring approximately 6 months to heal [31].

These complications led us to investigate alternative methods of identifying spermatozoa with a less impaired genome [32–34]. Indeed, this can be achieved to some extent with a standard sperm selection technique such as DGS, which appears to lower the level of SCF [35]. This well-established method, however, does not lower SCF below the normal threshold due to its centrifugation steps that generate reactive oxygen species (ROS) and its inability to select the most progressively motile spermatozoa [36–38]. Therefore, the known direct relationship between sperm progressive motility and chromatin integrity persuaded us to test a novel device that selects the most motile spermatozoa without having to centrifuge the specimen (Tables 1 and 2).

Our initial goal was to find a way to treat couples that had an impairment in male genomic integrity and a prior history of ART failure without resorting to invasive surgical techniques. The enhancement of motility, progression, and morphology we observed with the use of MSS was encouraging, but it was not as important as the remarkable reduction to SCF (Table 1), as has been confirmed by others [21]. These findings encouraged us to test this method of sperm sorting on 25 couples struggling with compromised SCF and persistent ART failure. The same effects of MSS on semen parameters were seen when preparing these semen samples for ICSI treatment. Intriguingly, we were able to achieve an average SCF lower than that of the 23 men we tested initially, even though this group of 25 had higher SCF compared with those men (Table 2).

In couples who had a fresh embryo replacement, MSS yielded spermatozoa with higher genomic competence as demonstrated by their ability to establish healthy pregnancies compared with cycles in which spermatozoa were processed in a standard fashion (Table 4). This was also the case for other groups that had spermatozoa selected using an apoptotic marker [39]. Interestingly, this was even true for couples who underwent PGT-A, as confirmed by the higher portion of euploid embryos that resulted in no term pregnancies (Table 6). In this cohort, MSS yielded a comparable proportion of euploid embryos and, for those couples who received a transfer by the time of this publication, all resulted in a viable pregnancy (Table 6). This remained true when we treated 16 couples with a history of recurrent implantation failure who, once treated by MSS, reported a clinical pregnancy rate of 58.8% (Table 8).

From these observations, it appears that a phenotypic selection of spermatozoa with the highest motility may yield a greater number of euploid embryos capable of resulting in a viable pregnancy. While it is difficult to comprehend how selecting spermatozoa with higher chromatin integrity would have any impact on the number of euploid embryos generated, this can only be explained by the type of nicks and breaks present within the chromatin [10, 28, 40]. When an SCF assessment was carried out with an alkaline Comet assay to detect single-strand DNA breaks and a neutral Comet assay to identify double-strand DNA breaks, the frequency of these chromatin defects was equivalent at about 50% in the fertile control as well as in oligo-astheno-terato-zoospermic patients [41]. Indeed, double-strand breaks may actually affect the chromosomal integrity of the male genome with consequent contribution to embryo aneuploidy, as has been seen in couples with idiopathic and recurrent pregnancy losses [42]. However, this will require further research utilizing an assay capable of specifically detecting this type of DNA damage, such as the neutral Comet assay.

## Conclusions

Within the quest for an optimal spermatozoon to inject during ICSI, this study proposes a non-invasive method to recover spermatozoa with superior genomic competence for ICSI treatment in men with high SCF in their ejaculate. These findings allow us to reconsider the relevant contribution of the male genome to the normal development of the conceptus. Most importantly, this study proposes a conservative approach to treat couples with recurrent ART and/or implantation failure without resorting to testicular biopsy.
